# Physical condition and perceived fatigue in post-covid patients: An observational descriptive study

**DOI:** 10.1590/1516-3180.2023.0167.R1.04122023

**Published:** 2024-03-08

**Authors:** Tamara Iturriaga, Fernanda Salazar-Pérez, Marta Casallo-Cerezo, Guillermo García-Pérez-de-Sevilla, Alicia Sosa-Pedreschi, Ignacio Diez-Vega, Marta Supervia, Olga Arroyo, Margarita Pérez-Ruiz

**Affiliations:** I Sport Sci, MSc, PhD. Professor, Department of Sports Sciences, Faculty of Sport Sciences, Universidad Europea de Madrid, Spain.; II Sport Sci, MSc, Professor. Department of Physiotherapy, Faculty of Medicine and Health Sciences, Universitat Internacional de Catalunya (UIC), Barcelona.; III MD, MSc, Physiatrist. Gregorio Marañón General University Hospital, Madrid, Spain.; IV PT, MSc, PhD. Professor. Department of Physiotherapy, Faculty of Sport Sciences, Universidad Europea de Madrid, Spain.; V Nutr Diet, MS, Professor. Department of Physiotherapy, Faculty of Sport Sciences, Universidad Europea de Madrid, Spain.; VI PT, MSc, PhD. Professor. Department of Nursing and Physiotherapy, Faculty of Health Science, Universidad de León, Ponferrada, Spain.; VII MD, MSc, PhD. Physiatrist. Gregorio Marañón General University Hospital, Gregorio Marañón Health Research Institute, Madrid, Spain; Cardiologist. Division of Preventive Cardiology Department, Cardiovascular Medicine Mayo Clinic (MN), Madrid, Spain; Faculty of Physical Activity and Sport Sciences, Universidad Politécnica de Madrid, Madrid, Spain..; VIII MD, MSc, PhD. Physiatrist. Gregorio Marañón General University Hospital, Gregorio Marañón Health Research Institute, Madrid, Spain.; IX MD, MSc, PhD. Profesor Titular. Grupo ImFine. Departamento de Salud y Rendimiento Humano, Facultad de Ciencias de la Actividad Física y el Deporte (INEF), Universidad Politécnica de Madrid, Madrid, Spain.

**Keywords:** COVID-19, Rehabilitation, Muscle Strength, SAR-Cov-2, Muscle mass, Fatigue syndrome

## Abstract

**BACKGROUND:**

Patients with severe coronavirus disease 2019 (COVID-19) often require hospital admission and experience sequelae such as chronic fatigue or low muscle mass.

**OBJECTIVE:**

To analyze the functional capacity of a cohort of patients with severe acute respiratory syndrome coronavirus 2 who required hospitalization.

**DESIGN AND SETTING:**

An observational descriptive study was conducted on post-COVID-19 patients referred to the Rehabilitation Department of Gregorio Marañón Hospital (Madrid, SPAIN).

**METHODS:**

Cardiorespiratory fitness, muscle strength, body composition, and perception of fatigue and dyspnea were analyzed. Furthermore, the existing correlations between clinical variables and physical conditions were analyzed.

**RESULTS:**

Forty-two patients who required hospital admission (80 ± 22.45 days) or intensive care unit (ICU) admission (58 ± 10.52 days) were analyzed. They presented with decreased strength, respiratory capacity, and moderate-to-severe perceived fatigue. Additionally, an inverse correlation was found between right-handgrip strength and days in the ICU, as well as the 6-minute walk test for women. Similarly, strength and fitness were negatively associated with perceived fatigue.

**CONCLUSIONS:**

Post-COVID-19 patients showed low muscle function and low levels of physical fitness associated with high perceived fatigue.

## INTRODUCTION

Coronavirus disease 2019 (COVID-19) is a viral infection caused by severe acute respiratory syndrome coronavirus 2 (SARS-CoV-2). The disease causes severe acute respiratory syndrome in 75% of patients and induces acute systemic inflammation,^1^ which requires 26% of patients admitted to the intensive care unit (ICU).^2^

Skeletal muscle-related symptoms are common in both acute illness and post-acute sequelae of COVID-19,^3^ among which chronic fatigue syndrome stands out, in part due to the significant reductions in muscle mass described after spending 7–20 days hospitalized in the ICU.^4^ In a recent study, patients admitted to the ICU for severe COVID-19 showed a 30% decrease in the cross-sectional area of the Rectus Femoris after 10 days.^5^ Many works have also described significant reductions in the maximum isometric strength of the Quadriceps and Biceps Femoris.^6^

These data are consistent with those of the first SARS-CoV-2 epidemic almost two decades ago. Patients hospitalized with this infection reported decreases of 32% in maximum isometric grip strength and 13% in the distance walked on the 6-minute walk test (6 MWT) several months after hospital discharge.^7,8^ In patients surviving critical illnesses other than SARS-CoV-2, who had suffered an extended hospitalization, significant muscle mass and strength losses were detected, possibly due to a reduction in protein synthesis, an increase in muscle breakdown, and systemic inflammation, the latter caused by the so-called cytokine storm, the same one caused by COVID-19, leading to a drastic rise in protein catabolism.^9^ These findings were reported even five years after patients had been released from the hospital.^10^ In this frame, ICU-acquired weakness developed above all in patients with more severe disease involvement,^11,12^ which are also those with the highest levels of inflammatory markers.^13^ Some of these biomarkers, such as C-reactive protein (CRP), interleukins (IL) such as IL-6, IL-1b, or tumor growth factor (alpha-TNF), can directly induce muscle proteolysis and decreased protein synthesis, especially when the inflammation comes from the lung.^14^ Other causes associated with muscle protein catabolism come from the nutritional status and rest associated with the disease.^3^

Performing early rehabilitation to recover muscle mass is of interest. To this end, physical strength exercise should be an essential component.^15^ In this sense, in a randomized clinical trial with 133 patients affected by SARS-CoV, in which a 6-week mixed strength and cardiorespiratory exercise program was carried out, with 4–5 weekly sessions of 60–90 minutes, the patients increased their isometric grip strength by 17%, shoulder flexion strength by 38%, and hip extension strength by 250%. Similar programs could benefit patients affected by COVID-19.^8^ However, currently, most studies on post-COVID-19 rehabilitation are quasi-experimental studies based on daily cardiorespiratory-type exercises, achieving significant improvements in the 6 MWT, which is associated with decreased perceived fatigue.^16^

Comparing patients admitted to the ICU for COVID-19 with patients admitted to the ICU for other reasons, the former presented higher levels of CRP and a direct correlation between inflammation, lung injury, and muscle breakdown.^17^ Patients with multiorgan involvement undergo higher muscle degradation. This systemic involvement is more frequent than that in other types of patients admitted to the ICU because intubated patients experience a more significant inflammatory response in the airways, which can cause inflammation in virtually every organ of the body.^18^

Another noteworthy fact is that elderly patients, who are the majority of patients severely affected by COVID-19, already have a situation of underlying muscle breakdown and systemic inflammation, even suffering from sarcopenia.^19^ Excess myokines and adipokines secreted by sarcopenic muscle stimulate oxidative stress and directly induce multiorgan damage, including skeletal muscle.^20^ Patients hospitalized for COVID-19 with pre-existing sarcopenia take twice as long to be discharged from the hospital and have a mortality rate eight times higher than those who do not suffer from it.^21^

## OBJECTIVE

Given the data previously expressed and the existing gaps regarding functional capacity and fatigue in this type of patient, the objective of the present study was to analyze and describe the functional capacity of a cohort of patients infected with SAR-Cov-2 requiring hospitalization during the 1^st^ and 2^nd^ waves of the pandemic in Spain. Additionally, differences according to sex and possible associations between body composition, physical condition, perception of fatigue, and length of hospitalization were analyzed.

## METHODS

A descriptive observational study was conducted. The data were obtained during the first six months of 2021 from the Rehabilitation Service. The study protocol was adjusted to the Helsinki Declaration Ethical Guidelines, whose last modification was written in 2013 and approved by the Clinical Research Ethics Committee of the hospital (Reference 26/2020 date 11/30/2020). The reference guide for manuscript preparation was the Strengthening the Reporting of Observational Studies in Epidemiology.^22^ All patients provided signed informed consent to participate in the study.

### Participants

Non-probabilistic convenience sampling was performed, where all patients referred by their pulmonologist or internist to the hospital's rehabilitation service were invited to participate. The inclusion criteria were as follows: (i) older than 18 years, (ii) having passed the SARS-CoV-2 infection with negative PRC, and (iii) admitted to the hospital between March 2020 and May 2021. The exclusion criteria were as follows: (i) patients with severe psychiatric illnesses, poor comprehension skills, or severe cognitive impairment; (ii) patients with contraindications for physical activity (acute myocarditis, uncontrolled arterial hypertension, arrhythmias, acute thrombosis, and infection); (iii) patients who revoked the informed consent signature due to not having an interest in participating.

Between March 2020 and May 2021, the Rehabilitation Service received 73 patients, 52 of whom were eligible for inclusion in the study. Of these 52 patients, five withdrew from the study. Therefore, 47 patients were included in the functional assessments and questionnaires, of which five did not complete the assessments; therefore, 42 patients were finally analyzed.

### Variables

#### Clinical History

Clinical variables were collected, such as age, date of hospital admission and discharge in the internal medicine ward, days of admission of those patients who were in the ICU; type of mechanical ventilation provided during hospitalization: (a) orotracheal intubation (OTI) or (b) non-invasive mechanical ventilation with high flow nasal cannula oxygen therapy (HFNOC); reason for admission: (a) due to bilateral SARS-CoV-2 pneumonia, (b) due to unilateral SARS-CoV-2 pneumonia, or (c) without pneumonia.

#### Assessment of cardiorespiratory capacity

An indirect test to estimate maximum oxygen consumption (VO2max) was performed to assess cardiorespiratory fitness using the 6 MWT according to the guidelines of the American Thoracic Society (ATS).^23^ The test consisted of walking as fast as possible (without running) for 6 min on a flat corridor over a distance of 20 m. Heart rate (HR) and oxygen saturation (SpO2) were recorded with a pulse oximeter (Agptek. Brooklyn, USA) at four times: (1) at rest just before starting the test, (2) immediately at the end of the test, (3) two minutes after recovery began, and (4) five minutes after recovery began.

#### Assessment of muscle function

Two tests were used to estimate the neuromuscular performance: 1) Manual Handgrip (Jamar. Chicago, USA) in the upper body, the patient was seated on a chair with the elbow at 90°, the dynamometer was pressed with maximum force, and the value obtained was recorded. This procedure was performed twice, and the average values for both the right and left hands were recorded. In addition, the patient's dexterous hand was recorded.^24^ 2) The Chair Stand Test (30sCST) in the lower body: the patient was seated in a chair to stand and sit as many times as possible in 30 s, ideally without support, unless the patient needed it. The type of support provided was recorded.^25^

#### Body Composition Assessment

Body composition data such as body weight (kg), body mass index (BMI) (kg/m2), fat mass (%), and fat-free mass (kg) were measured using a bioelectrical impedance balance (Tanita Ironman InnerScan BC-545N. Amsterdam, the Netherlands). Furthermore, the waist-to-hip ratio (WtHr) was measured using a tape measure (Rosscraft Anthro Tape. St. Paul, MA, USA) to assess the risk of developing cardiovascular diseases. This index is derived from the ratio between the waist circumference and hip circumference in centimeters, with reference values for both men and women.^26^

#### Self-reported questionnaires

Each patient was given five questionnaires: 1) Rapid Assessment Physical Activity (RAPA), measuring the level of physical activity, consisting of nine items, seven of which sought to determine if the patient met the minimum exercise recommendation^27^ (30 minutes or more at least five days a week), assessing the frequency and intensity at which they performed these activities, and two additional items measuring whether the patient performed flexibility and strength exercises.^28^ 2) The Brief Fatigue Questionnaire presents a numerical rating scale from 1 to 10, with 10 being the worst fatigue imaginable. It is divided into four items that rate the degree of fatigue perceived in 24 hours and six items that rate how much this fatigue interferes in different life situations.^29^ 3) The SARC-F Scale, which assesses muscle weakness by determining the degree of sarcopenia of the patient, uses five items that rate the difficulty that the patient presents in performing actions that involve both the stability and the strength of their upper and lower limbs. If the total score was equal to or greater than 4 points, the presence of sarcopenia was considered.^30^ 4) Dyspnea analog scale, in which the patient must assess the perception of breathlessness graphed in drawings and numbers, where 0 = absence of breathlessness except when exercising; 1 = I choke when walking too fast or going up a slight hill; 2 = I choke when walking on the flat at the same pace as other people my age or I have to stop to rest; 3 = The choking forces me to stop before 100 meters or after a few minutes when walking on a flat ground; 4 = I choke when making daily efforts such as getting dressed or leaving the house, and I have to stop.^31^

#### Assessment protocol

During consultation with a rehabilitation doctor, the patient signed an informed consent form to participate in the study. Subsequently, the patient was scheduled to undergo physical tests and complete the questionnaires. On the appointment day, the patient answered self-reported questionnaires and his body composition was assessed, followed by muscle function tests, ending with the cardiorespiratory fitness test.

### Statistical analysis

Data were collected in one notebook per patient and codes were assigned. After data collection, a database was created for further analysis. First, the Shapiro–Wilk test was performed to determine whether the variables were parametric or nonparametric. Descriptive statistics were performed, presenting data of mean and standard deviation (Mean ± SD) of all variables that determine the characteristics of the sample. Furthermore, for the following variables: (i) reason for admission, (ii) mechanical ventilation provided, (iii) estimated maximum oxygen consumption, (iv) Waist-to-Hip Ratio, (v) BMI, (vi) dyspnea scale, (vii) fatigue level, and (viii) sarcopenia frequency, a test was performed to determine the distribution of the sample in percentages (%) by category. A chi-square (X2) test was performed to analyze differences in data distribution between sexes. Finally, to analyze the correlations among the clinical variables, physical condition, muscle function, and perceived fatigue, the Spearman test (rs) was performed, as all variables were non-parametric. A value of coefficient rs smaller than 0.40 is considered weak correlation, 0.40–0.69 moderate correlation, and > 0.70 strong correlation. All analyses were performed using the Statistical Package for the Social Sciences (SPSS 20.1 for Windows, IBM, Armonk, New York, United States).

## RESULTS

Forty-two post-COVID-19 patients (mean age 59.9 ± 10.06) derived from the rehabilitation service were analyzed. The mean days admitted to the ICU were 58 ± 10.52 days and 80 ± 22.45 days in admission to the ward. When comparing the results according to sex, the percentage of men admitted to the ICU was much higher than that of women (85.7% and 47.6%, respectively). Descriptive results are shown in Table 1, separated by anthropometric and physical condition variables represented as means and standard deviations.

**Table 1:** Characteristics of the sample

		Mean ± SD Men n = 21	Mean ± SD Women n = 21	P value
**Anthropometric Variables**				
	Age (years)	60.95 ± 9.56	58.9 ± 10.68	0.51
	Weight (kg)	91.08 ± 17.91	80.9 ± 20.84	0.09
	Height (cm)	170.85 ± 6.61	158.33 ± 5.44	0.001
**Body Composition**				
	BMI (kg/m2)	31.03 ± 4.79	32.08 ± 7.25	0.58
	Fat mass (%)	31.67 ± 7.24	41.35 ± 8.3	0.001
	Fat-free mass (kg)	58.54 ± 9.92	43.63 ± 6.27	0.001
**Physical Condition and Functional Capacity**				
	6MWT Distance (m)	453.90 ± 111.55	388 ± 90.66	0.04
	6MWT VO_2_ max estimated (ml/kg/min)	27.83 ± 5.07	24.51 ± 7.36	0.09
	right handgrip (kg)	25.31 ± 9.94	15.21 ± 6.85	0.001
	relative right handgrip (kg/kg weight)	0.30 ± 0.11	0.19 ± 0.11	0.004
	left handgrip (kg)	23.34 ± 9.11	13.2 ± 6.79	0.001
	Relative left handgrip (kg/kg weight)	0.28 ± 0.10	0.19 ± 0.10	0.006
	Sit to Stand Test (n° repetitions)	12.09 ± 5.75	9.66 ± 5.54	0.17

P value: P < 0.001; Data are presented as Mean ± SD; 6 MWT = 6 Minutes’ Walk Test; BMI = body mass index. The Mann–Whitney U test was performed to analyze the differences between female and male participants.

Comparing male and female participants, there were significant differences in the percentage of fat mass (P = 0.001), fat-free mass (P = 0.001), and right handgrip, both in kg and kg relative to body weight (P = 0.001 and 0.004, respectively), and also in the left handgrip, both in kg and kg relative to body weight (P = 0.001 and P = 0.006, respectively) (Table 1).

The sample distribution by category was analyzed for the following variables: clinical variables and (i) reason for admission, where 90.5% of men and 85.7% of women were admitted for bilateral pneumonia; (ii) Types of supplied ventilation. Regarding body composition variables, we observed that 90.4% of men and 80.9% of women were overweight or obese, according to BMI. Concerning the cardiovascular risk index (Waist-to-Hip Ratio), over 70% of the participants were categorized as high risk. Physical fitness levels were low in both men (57.1%) and women (76.2 %). For levels of physical activity (RAPA), most of the participants were categorized as low-active (48% of men and 14% of women) or moderately active (38% of men and 57% of women). According to self-reported questionnaires, only 19% of men and 47.6% of women had sarcopenia. They did not claim significant dyspnea, and most of the cohort analyzed claims that perceived fatigue as moderate (57.1% men and 33.3% women) to severe (19% men and 47.6% women). The data are presented in detail in Table 2.

**Table 2:** Distribution by sex of clinical variables, body composition, physical condition, and self-reported questionnaires

		Men n = 21	Women n = 21	X^2^
**Reason for admission**				
	Unilateral Pneumonia	4.8%	0.0%	0.36
	Bilateral Pneumonia	90.5%	85.7%	
	Others	4.8%	14.3%	
**Type of ventilation**				
	MV. OTI	61.9%	33.3%	0.07
	MV. HFNCOT	19%	14.3%	
	Without MV	52.4%	19%	
**Body composition: body mass index**				
	Normal	9.5%	19%	0.60
	Obesity	33.3%	23.8%	
	Overweight	57.1%	57.1%	
**Cardiovascular risk (Waist-Hip Ratio)**				
	Low risk	19%	28.6%	0.46
	High risk	81%	71.4%	
**Physical condition: estimated maximal oxygen consumption**				
	Low	57.1%	76.2%	0.19
	Normal-High	42.9%	23.8%	
**Rapid Assessment Physical Activity (RAPA)**				
	Sedentary	0%	10%	0.08
	Little active	48%	14%	
	Moderately active	38%	57%	
	Active	14%	19%	
**Dyspnea Scale**				
	0	0%	14.3%	0.07
	1	57.1%	33.3%	
	2	33.3%	42.9%	
	3	9.5%	0%	
	4	0%	9.5%	
**Perceived fatigue**				
	Mild	23.8%	19%	0.13
	Moderate	57.1%	33.3%	
	Severe	19%	47.6%	
**Sarcopenia**				
	Yes	19%	47.6%	0.05
	No	81%	52.4%	

Data are presented as percentages. MV. HFNCOT: mechanical ventilation. High-Flow Nasal Cannula Oxygen Therapy OTI: mechanical ventilation. orotracheal intubation; The dyspnea scale ranges from 0 to 4, where "0" does not present dyspnea and "4" present dyspnea with daily living activities.

Significant associations were observed between clinical variables and physical conditions. The relative strength of body weight of the right hand showed an inverse correlation with the days of ICU admission for men (rs = 0.156), unlike women, where no correlation was seen between them (rs = 0.00059). However, we found an inverse association between travel distance in the 6 MWT and ICU admission days (rs = 0.165), but not in men (rs = 0.004).

Regarding perceived fatigue, an inverse trend was observed, although not significant, with the right handgrip relative to body weight and distance traveled in the 6MWT in both cases; thus, the higher the perceived fatigue, the lower the force of the handgrip and the lesser the distance traveled in the 6 MWT. Additionally, non-significant but clinically relevant trends were observed for fat mass, ICU admission days, and perceived fatigue. Participants with the highest fat mass percentage reported the greatest increase in perceived fatigue and were admitted to the ICU for more days.

## DISCUSSION

COVID-19 presents a tremendous challenge for the global population, particularly at the clinical level. If the disease is overcome, one of the most severe problems is physical deterioration that patients experience after medical discharge, mainly due to the extended time they are admitted with reduced mobility and systemic inflammation caused by the virus itself. This was reflected in the cohort of post-COVID-19 patients analyzed in this study; both men and women had a significant deterioration in body composition, with a very high percentage of fat mass and a mean body mass considered as "obesity" by the World Health Organization.^32^ Furthermore, there was a high Waist-to-Hip Ratio in both men and women, which reflected an increased risk of cardiovascular disease in both.^32^ In this line, it is essential to note that women had worse body composition than men, with statistically significant differences in fat mass percentage and fat-free mass. In addition, only 14% of men and 19% of women in this study declared themselves physically active.

In line with the study by Eksombatchai et al., the physical condition assessed with the 6 MWT reflected a low cardiorespiratory capacity, possibly due to lung involvement caused by the virus and the prolonged downtime produced by hospital admission.^33^ Furthermore, the present study participants presented with very low handgrip values, which pose a risk of all-cause mortality.^34,35^ These findings are in line with studies reported on the SARS-CoV-1 epidemic nearly two decades ago.^7,8^

According to numerous observational studies, one of the main sequelae found in post-COVID-19 patients is the presence of persistent fatigue, which, in many cases, persists even months after hospital discharge.^36,37^ In the present study, a high percentage of patients reported experiencing moderate-to-severe fatigue in the tasks of daily living. In addition, the perception of fatigue was negatively associated with muscle strength measured by handgrip; therefore, patients who had less relative strength in the right hand reported more significant fatigue, which was more relevant to this association in women. Similarly, a study analyzing perceived fatigue after post-COVID-19 in patients with type 2 diabetes established the same negative relationship between grip strength and perceived fatigue.^38^ In the present study the same association was found concerning the distance achieved in the 6 MWT test. Patients who perceived significant fatigue achieved shorter distances in the test.

In addition to significant pulmonary involvement and its consequences, these patients are admitted for an average of 80 days, which is highly detrimental to muscle health. According to Puthucheary et al., after seven days in bed, 10% of muscle mass is lost.^17^ This results in muscle mass brings with it a loss of strength, muscle atrophy, changes in muscle fibers (from type I to type II), worse oxidative capacity, loss of mitochondrial capacity,^39^ and a considerable risk of disability.^40^ All these findings justify that these patients perceived more significant fatigue. Similarly, we found negative associations between the total number of days admitted to the ICU and lower grip strength, in addition to shorter distance in the 6 MWT. In line with these findings, other studies on post-COVID-19 patients have also found negative correlations between the severity of affectation and muscle weakness.^11,12^

In addition, a positive association was observed between body fat percentage, days admitted to the ICU, and the level of perceived fatigue. Participants with greater adiposity had longer ICU stays and higher perceived fatigue. According to a recent systematic review, obesity is a risk factor for the development of a more severe form of COVID-19 because this infection is highly inflammatory, and these patients already have high levels of systemic inflammation.^41^

### Limitations of the study

Despite presenting clinically relevant findings, this study has some limitations. First, as this was an observational study, causal relationships could not be established. There is no certainty whether patients with a lower initial physical condition required a longer hospitalization time and developed a more severe form of the disease or whether it was the fact that they had a longer hospital admission time or were admitted to the ICU, which led to a more significant functional impairment. Additionally, the wide age range of the participants may have influenced the results. Finally, it would be interesting to compare these results with those of a sample of ICU patients without COVID-19.

## CONCLUSION

The post-COVID-19 participants were mostly obese and sedentary older adults who presented with low muscle function and low levels of physical condition associated with moderate-to-severe fatigue. Male participants required ICU admission more frequently than female participants. Obesity was associated with increased perceived fatigue and a longer ICU stay.
